# Properties of Titanium Zirconium Molybdenum Alloy after Exposure to Indium at Elevated Temperatures

**DOI:** 10.3390/ma15155270

**Published:** 2022-07-30

**Authors:** Florian Metzger, Vincent Rienzi, Christopher Mascetti, Tri Nguyen, Siddha Pimputkar

**Affiliations:** Department of Materials Science and Engineering, Lehigh University, Bethlehem, PA 18015, USA; fsm217@lehigh.edu (F.M.); rienzi@umail.ucsb.edu (V.R.); cpmascetti@gmail.com (C.M.); tring@stanford.edu (T.N.)

**Keywords:** TZM alloy, mechanical properties, indium, intermetallic, corrosion, DTA, high pressure

## Abstract

Titanium zirconium molybdenum (TZM) is a high strength at high temperature alloy with favorable properties for use in high temperature structural applications. Use of TZM in high pressure, gas-containing autoclave systems was recently demonstrated for the ammonothermal method. Use of indium (In) in the system is desired, though there is a general lack of literature and understanding on the corrosion and impact of In on the mechanical properties of TZM. This study reports for the first time the mechanical properties of TZM after exposure to metallic In at temperatures up to 1000 °C. Static corrosion testing of TZM in In were performed at 750 °C and 1000 °C for 14 days. A microstructure analysis was performed suggesting no visible alteration of the grain structure. Differential thermal analysis (DTA) was performed to investigate compound formation between In and the primary constituents of TZM yielding no measurable reactions and hence no noticeable compound formation. X-ray energy dispersive spectroscopy (EDS) line scans across the TZM-In interface revealed no measurable mass transport of In into the TZM matrix. These results were confirmed using X-ray diffraction (XRD). Given the apparent inertness of TZM to In, mechanical properties of TZM after exposure to In were measured at test temperatures ranging from 22 °C to 800 °C and compared to unexposed, reference TZM samples from the same material stock. Tensile properties, including ultimate tensile strength, yield strength and total elongation, were found to be comparable between In-exposed and unexposed TZM samples. Impact fracture toughness testing (Charpy) performed at temperatures ranging from −196 °C to 800 °C showed that TZM is unaffected upon exposure to In. Tensile testing indicated ductile behavior at room temperature (slow strain rate) whereas impact testing (high strain rate) suggested a ductile to brittle transition temperature between 100 °C and 400 °C. Given these results, TZM appears to be a promising candidate for use as a force bearing material when exposed to In at high temperature.

## 1. Introduction

Titanium zirconium molybdenum (TZM) is a molybdenum-base alloy engineered for high strength at high temperatures. It exhibits a low thermal expansion coefficient, high thermal and electrical conductivity and excellent thermal shock resistance [1,2,3,4,5]. TZM B387 type 364 nominally contains 0.4–0.55 wt% titanium (Ti), 0.06–0.12 wt% zirconium (Zr), 0.01–0.04 wt% carbon (C), and balance molybdenum (Mo) [6]. TZM exhibits greater strength and creep resistance compared to unalloyed Mo at elevated temperatures due to precipitation strengthening via formation of Ti and Zr carbides [7,8].

While TZM exhibits a remarkable corrosion resistance to corrosion by molten metals and electrochemical processes, it oxidizes at temperatures above 400 °C evolving volatile molybdenum oxides [9,10,11,12,13]. In recent years, many studies were conducted to improve upon the oxidation resistance of TZM primarily by adding coatings to the exposed surfaces [14,15].

Currently, TZM is used in applications at high temperatures in the nuclear, aerospace and electronics industries due to suitable mechanical properties under operating these conditions [1,2,3,4,5,16,17]. Recently, TZM’s application as the pressure-retaining material for pressure vessels containing supercritical ammonia at temperatures up to 900 °C and pressures up to 100 MPa was demonstrated for the first time [18,19]. 

Use of supercritical ammonia as a solvent for synthesis of nitrides, an approach known as the ammonothermal method, has taken on a prominent role in synthesis of large boules of single crystal gallium nitride (GaN) [20,21]—a wide-band gap semiconductor which has garnered significant interest in recent years due to its applications in visible and UV light emitters and power electronics [22,23,24]. Synthesis of its ternary alloys with indium (InGaN, AlInN) along with large, single crystal synthesis of pure indium nitride (InN) are formidable challenges and have yet to be demonstrated using this technique. 

While growth of sub-mm-sized hexagonal InN crystals was recently demonstrated using this approach, cumbersome ceramic liners were needed to protect the indium-incompatible nickel-chromium superalloy autoclave wall [25]. Use of TZM, a potential indium-resistant material, as the autoclave wall material would prove transformative for single crystal synthesis of indium-containing nitrides due to elimination of numerous hurdles associated with the use of sealed ceramic liners. 

Literature review yields little information on the high-temperature behavior of TZM when exposed to indium (In). Johnson [26] investigated TZM as the pipe material for liquid-In cooling for a 438 h (18.25 days) exposure at 1000 °C, although the reported results are inconclusive. The initial roughness of the tested TZM tube was too high to measure dissolution or corrosion of the material post-In exposure. X-ray analysis of the In post-run showed large amounts of dissolved Mo (≥1000 ppm) and Ti (200–500 ppm). This dissolution is in agreement with the associated binary phase diagrams of In-Mo and In-Ti [27]. No data was reported on the impact In had on the mechanical properties of TZM pipe. 

Literature also does not provide significant insight into the interactions between In and zirconium carbide (ZrC) or titanium carbide (TiC) other than suggesting a poor wettability of TiC by In up to 847 °C [28].

Given the current state of literature, it is not possible to reliably understand or predict the behavior and suitability of TZM as a structural material when exposed to In. While it is known that In and Mo do not form any intermetallic compounds based on the binary phase diagram of In-Mo [27], TZM critically relies on the presence of carbides to enhance its mechanical properties. As such, a dedicated study is required to investigate the possible negative impact In could have on the mechanical properties of TZM due to possible reactions occurring within the bulk or grain boundaries of the material which would negatively impact the mechanical behavior of the material. This study investigates possible chemical interactions of In with TZM and resulting impact on mechanical properties at high temperature for the first time. Chemical reactions between In and Mo, In and TiC, and In and ZrC were investigated using differential thermal analysis (DTA). Mechanical properties were determined by exposing TZM tensile and Charpy samples to In at 750 °C or 1000 °C for 14 days and then analyzing the samples at cryogenic, room, and elevated temperatures for their yield strength, ultimate tensile strength, elongation at failure, area reduction at failure, and fracture toughness. Indium-exposed metallographic samples were analyzed for microstructural changes using scanning electron microscopy (SEM) and light optical microscopy (LOM). Compositional changes to the samples were analyzed using X-ray energy dispersive spectroscopy (EDS). Indium-exposed TZM samples are compared to unexposed, reference TZM samples from the same material stock along with available literature data to provide a reference for anticipated TZM behavior when not exposed to In.

## 2. Materials and Experimental Procedures

### 2.1. Chemical Reactivity and Compound Formation

DTA measurements were conducted to measure material interactions and reactivity of In with Mo, In with TiC, and In with ZrC. A Netzsch STA 409C (NETZSCH-Gerätebau GmbH, Selb, Germany) device using constant heating and cooling rates of 3 °C min^−1^ was utilized. The samples were heated from room temperature to 1000 °C with an isothermal hold of 20 min and cooled down to room temperature. Prior to running the experiments, the measuring chamber was evacuated and back filled with purified argon (99.998%) three times. The chamber was purged continuously with an argon gas flow of 10 cm^3^ min^−1^. 

The interaction between In (99.999%, Indium Corp., Clinton, NY, USA) and the Al_2_O_3_ crucible was measured to record a baseline. The tear-shaped In droplets were cut into 10–15 pieces and directly inserted into the crucible.

For investigating reactions between In and carbides, tear-shaped In droplets were cut into 10–15 pieces, mixed with the respective carbide powders in atomic ratios of In: TiC (99.5%, Alfa Aesar, Ward Hill, MA, USA) and In: ZrC (99.5%, Alfa Aesar) of approximately 2:1, and then filled into an Al_2_O_3_ crucible. 

Interactions of Mo with In were investigated by filling a smaller Mo crucible (Moltun International, Cheyenne, WY, USA) with In, placing it in an Al_2_O_3_ crucible while compacting an Al_2_O_3_ (99.7%) powder bed around it to ensure good thermal contact between all elements in the system.

As the differential reference, an Al_2_O_3_ crucible filled with 500 mg of Al_2_O_3_ powder (99.7%) was used for all measurements. 

All crucibles were used with lids made of their respective material to prevent the evaporation of reactants, primarily In as it has a vapor pressure of 5.47 Pa at 1000 °C. 

X-ray diffraction (XRD) measurements were conducted using a PANalytical Empyrean diffractometer with a copper X-ray tube emitting at a wavelength of 1.541 Å.

### 2.2. TZM Sample Preparation

Commercially available TZM rods (ASTM B387 Type 364, stress relieved) were obtained from Ed Fagan Inc., Franklin Lakes, NJ, USA (Heat#: SZ40058, DIA: 12.7 mm, Label: TZM 1) and Eagle Alloys Corporation, Talbott, TN, USA (Heat#: TZM170501, DIA: 16.88 mm, Label: TZM 2) and used as starting materials. The chemical compositions of the TZM raw materials are given in Table 1.

### 2.3. Metallographic Samples

Metallographic samples were prepared and analyzed to investigate chemical and physical changes in the vicinity of the TZM-In interface using optical and chemical techniques. Samples were obtained by cutting 3–5 mm thick slices across the transversal oriented grains of the TZM 1 rod and then cutting them into halve along the longitudinal grain direction to obtain half circle cylindrical pieces. Samples were placed onto a graphite cloth which was placed into a round graphite crucible. Only one sample was loaded into the crucible per run. The half discs were placed such that they were sitting on the round side with the cut surface facing upwards. Enough In was added to fully submerge the sample in liquid In and no restraining mechanisms were needed as TZM has a higher density compared to In. 

The graphite crucible was placed into a tube furnace (Thermcraft, Inc. XST-4-0-12-1V2-F0, Winston Salem, NC, USA) with a ceramic tube made of Al_2_O_3_ (99.7%). The crucible containing the metallographic samples was co-loaded in the furnace with the crucible for the tensile sample (see Section 2.4). 

Before heating the furnace, the tube was evacuated and back filled with nitrogen (99.9995%, excluding Ar) three times. A purge flow of nitrogen was set to 90 cm^3^ min^−1^ during the runs. The heating rate of the furnace from room temperature to 750 °C or 1000 °C was 5 °C min^−1^. The samples were soaked at the desired temperature for 14 days. The mass of the samples and crucible components, were measured using a scale (Mettler College150, d = 0.1 mg) pre- and post-exposure.

Post run the metallographic samples were cleaned. The half discs were placed on a graphite cloth on a Al_2_O_3_ boat and placed back into the furnace. After three pump-purge cycles, the furnace was heated to 350 °C under nitrogen flow to melt the In off the sample. The remaining trace amounts of excess In on the surface were gently removed using an ethanol-soaked lint-free wipe by hand. Ethanol can be used to selectively remove In without dissolving Mo [29]. Care was taken to ensure only In was removed and no TZM material.

Samples were prepared for the metallographic analyses by grinding and polishing according to standard procedures for Mo and Mo-alloys [30] using different grades of SiC grinding papers up to a 1200 grit. Polishing was performed initially using a 1 µm Al_2_O_3_ slurry followed by a 0.3 µm Al_2_O_3_ slurry. The final polish was performed using a 0.05 µm SiO_2_ suspension on a polishing cloth.

Microstructural characterization was conducted via scanning electron microscopy (SEM). An FEI Scios dual beam FIB/SEM, operated at an accelerating voltage of 30 kV, was used to collect secondary electron (SE) and backscattered electron (BSE) images. X-ray energy dispersive spectroscopy (EDS) was conducted using a 30 kV electron beam. EDS line scans were collected using an EDAX Octane Elite silicon drift detector.

The longitudinal and transversal planes of the TZM specimens were polished and etched in a solution of 15 g K_3_Fe(CN)_6_, 2 g NaOH, and 100 mL H_2_O for 10 s. Microstructures were observed using a light optical microscope (LOM) (Zeiss Axiovert 40 MAT).

### 2.4. Tensile Samples

After investigation the physiochemical properties of the TZM-In interface mechanical properties were characterized to gain insight in mechanical behavior of TZM post In exposure. Standard small-size tensile specimens were machined by Ed Fagan Inc from TZM 1 according to ASTM E8 (#3) with a gauge length of 1 inch and a diameter of 0.25 inch. Tensile tests were performed in accordance with ASTM E8 which allow for no more than a 1% total error. Samples were tested by straining in the longitudinal orientation of the raw material.

**Figure 1:**
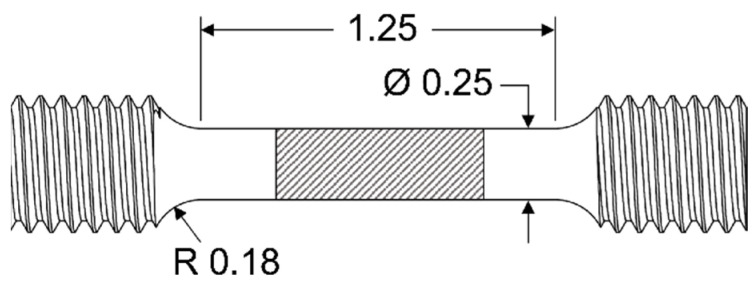
Schematic of tensile testing samples with nominal dimensions in inch. Hatched region illustrates the contact area of the TZM surface with In.

To expose the samples to In, specimens were individually placed within a custom graphite crucible (99.99%) designed to ensure contact of liquid In along the gauge region and not the grips. Figure 1 shows a schematic of the tensile sample geometry and area which was exposed to In (hatched region).

Placement of the samples into the tube furnace and post-run In-removal followed the same procedure as the metallographic samples described previously. Indium exposure runs were held at 750 or 1000 °C for 14 days.

Post-run the TZM tensile samples were pulled at −196 °C, 22 °C, and 800 °C at Westmoreland Mechanical Testing & Research Inc (WMTR). Reference tensile samples, which were not exposed to In, were machined from the same TZM 1 rod and tested at 800 °C by WMTR. The testing was performed at a strain rate of 0.005 min^−1^. All tensile tests at 800 °C were performed under a protective argon atmosphere.

### 2.5. Fracture Toughness Testing

Standard size Charpy V-notch impact testing samples (55 mm × 10 mm × 10 mm) according to ASTM E23 were machined by WMTR using TZM 2. Six samples were co-loaded into a graphite crucible with a lid for exposure runs in In. Custom made graphite sample holders were used to separate the samples to ensure complete contact of In and TZM. The samples were oriented with their notches up. Enough In was added to completely submerge the TZM specimens before placing them into the tube furnace. It was verified post-run via visual inspection that the In completely penetrated the notches with no gap between the In and the TZM notch tip.

Placement of the samples into the tube furnace and post-run In-removal followed the same procedures previously described for the metallographic samples. Soaking temperature was set to 750 °C for 14 days inside the tube furnace.

Charpy impact testing of the exposed and reference (unexposed) samples was performed at WMTR in the L-R direction according to ASTM notation [31]. Pairs of exposed and unexposed samples were tested at −196 °C, 22 °C, 100 °C, 400 °C, and 800 °C. The 800 °C tests were heated in a furnace with argon atmosphere immediately before testing.

## 3. Results and Discussion

This section is divided into four sections. Each section presents and discusses experimental results relevant to a particular topic of investigation. The first section discusses the microstructure of the samples. The second section investigates the chemical reactivity and compound formation of In with Mo and In with carbides using DTA. The third section investigates chemical changes to the samples, with a particular focus on In diffusion into the sample from the TZM-In interface. The fourth section investigates mechanical properties including yield strength, ultimate tensile strength, elongation at failure, area reduction at failure, and fracture toughness.

### 3.1. Metallographic Characterization

After performing post-run treatments on the samples, no signs of coloring or dissolution of TZM is visible in the SEM or LOM micrographs. The metallographic sample gained 0.009% mass after the exposure run. Assuming the maximum amount of Mo dissolution into the liquid In based on the phase diagram, a mass loss of 0.27% would be anticipated [27,32]. As no visible signs of dissolution or etching of the TZM surface were present, the lack of mass loss is attributed to insignificant Mo dissolution and not a dissolution of Mo combined with a substantial In uptake via diffusion. The observed mass increase of 0.009% can be explained by trace amounts of In on the surface of the half disc after cleaning. Residual In on the surface was confirmed using EDS (Section 3.3). The sensitivity of these measurements cannot exclude the possibility that small amounts of Mo or carbides leached into the In melt.

**Figure 2:**
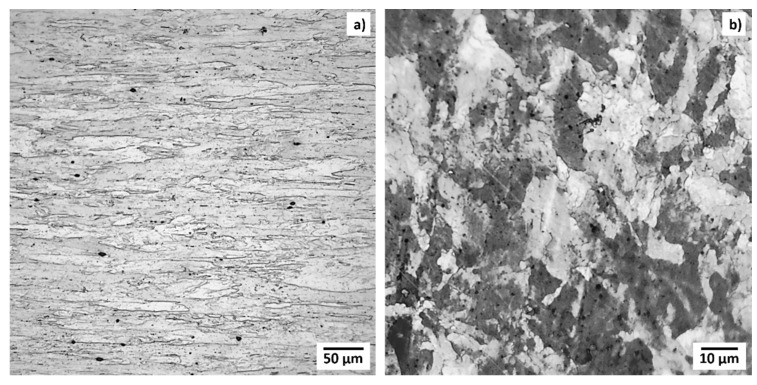
Optical images of the grain microstructures of the metallographic samples of TZM in the center of the rod post In exposure: (**a**) longitudinal plane, and (**b**) transversal plane. Black precipitates are mostly composed of Zr and O.

Polished cross-sections were etched to visualize grain boundary contrast. Figure 2 shows the microstructure of the TZM sample slices post exposure to In in (a) longitudinal and (b) transverse direction. Metallographic investigations exposed the typical microstructure of highly deformed grains. The TZM grains are elongated along the rod axis (longitudinal) direction with high grain aspect ratios of approximately 10–15. Typical grain sizes along the longitudinal axis of specimens were up to 150–200 µm, whereas transverse dimensions were typically below 15–20 µm with smaller subgrains in the center of the rod. Elongated grains were observed over the whole cross section of the TZM rod. However, the grains became more and more needle-like from the center towards the edge of the sample. Grains at the edge of the rods exhibit a higher aspect ratio compared to grains in the center. Observed grain size, shape, and orientation agree with literature analyzing common TZM in rod format [3,33]. Optical investigations of the interface of TZM and In did not reveal any interactions of the two metals. No evidence of grain boundary diffusion, deterioration or transformation was visible.

**Figure 3:**
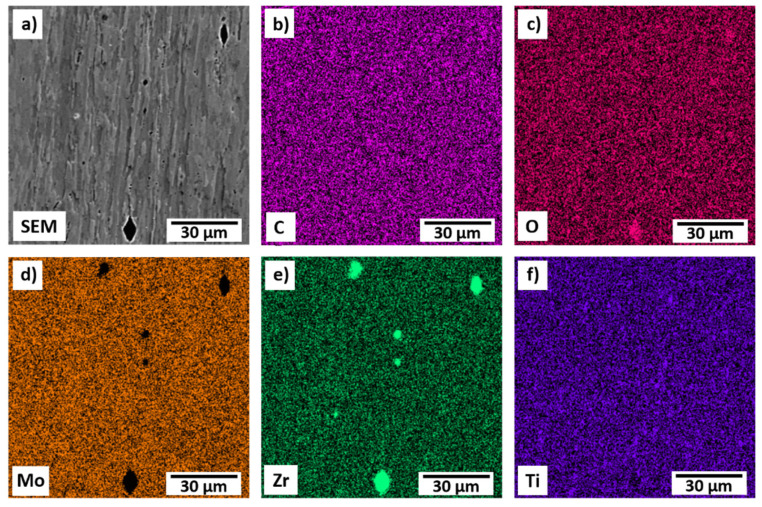
EDS map of the surface of TZM after polishing and etching characterizing black particles as ZrO_2_.

Post-polishing and etching round particles, typically smaller than 10–15 µm, were found on the surface of the sample. EDS mapping (Figure 3) determined the composition of the precipitates to be predominantly Zr and O. This finding has been previously described in the literature [3]. ASTM standards for powder metallurgical TZM (B 386 and B 387) allow higher concentration of O in the final product. Remaining Ti and Zr which did not form their respective carbides can react with residual O to TiO_2_, ZrO_2_, or its mixtures to form precipitates which are principally located at or near grain boundaries. The identified ZrO_2_ particles are typical artifacts from the manufacturing process and are unavoidable [11].

### 3.2. Chemical Reactivity and Compound Formation

Chemical reactions or phase changes typically involve the absorption (endothermic) or release of energy (exothermic). By measuring and comparing the temperature of a sample undergoing transformation relative to an inert, reference sample, the occurrence of a reaction or phase transformation can be identified. DTA is a common and sensitive tool used to investigate this behavior and analyzing the first derivative offers a clear indication on the onset of a reaction.

While it would be convenient to directly expose In to TZM and determine any compound formations or chemical reactions, it is anticipated that the thermal energy exchange of In with trace amounts of TiC and ZrC in the grain boundaries of TZM would be too small to be measurable by DTA. As a result, measurements with pure Mo and powders of pure TiC and ZrC were individually exposed to In. To maximize the surface area contact between In and the carbide powders, the powder was intermixed with In.

**Figure 4:**
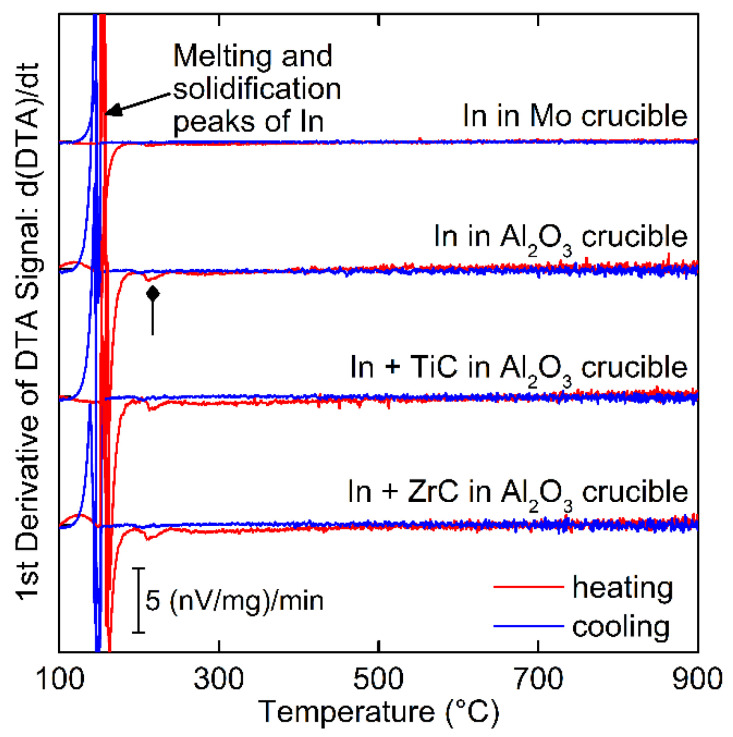
First derivative of DTA signals (nV mg^−1^ min^−1^) for heating and cooling curves of In exposed to Mo (top trace), Al_2_O_3_ (2nd trace from top) and for In with TiC (3rd trace) or ZrC (4th trace) in Al_2_O_3_ crucibles. Arrow at ~157 °C marks melting and solidification peaks. Peaks at ~215 °C occur during the heating cycle whenever In is exposed to Al_2_O_3_ (diamond head arrow).

The chemical reactivity between In and either Mo, TiC, or ZrC is reported in Figure 4 as the first derivative of the measured DTA curves of the heating and cooling cycles. A reference sample including exposure of just In to the Al_2_O_3_ crucible is also provided. All DTA traces exhibit an anticipated large peak around the melting temperature of In at 157 °C.

The In-Mo sample (“In in Mo crucible”) yields a flat trace after melting of In indicating no chemical interactions or structural changes occurred in the system. This result is anticipated and confirms the binary phase diagram of In-Mo [27]. Note that the enthalpy of dissolution of In into Mo is presumed to be too small to be measurable using DTA.

Due to insufficient free volume in the Mo crucible, exposure of In to the carbides had to be performed in a larger Al_2_O_3_ crucible. Pure In was measured in an Al_2_O_3_ crucible (“In in Al_2_O_3_ crucible”) to obtain a baseline for comparison for the In and carbide runs in an Al_2_O_3_ crucible (“In + TiC in Al_2_O_3_ crucible”, and “In + ZrC in Al_2_O_3_ crucible”).

The heating and cooling curves for the three samples are shown in Figure 4 and exhibit the anticipated melting and solidification peaks of In along with no statistically relevant peaks above 250 °C. Curves obtained from measurements with In in direct contact with Al_2_O_3_ were noisier compared to the measurement with the Mo crucible, presumably due to the closer proximity of the sample to the thermocouple and the lack of additional thermal mass of the Mo crucible which would smoothen the mass-normalized signals.

A broad peak is observed for all three traces at 215 °C (see diamond head arrow Figure 4), yet only for heating and never for cooling cycles. Given the presence of the peak in the reference sample (only In and Al_2_O_3_), an extensive literature survey was performed to identify the possible origin of this peak. No reactions are known to occur between In and Al_2_O_3_ around 215 °C, though a higher temperature compound (AlInO_2_) is known to exist, though unlikely to form at this low temperature [34]. Post-run, the In sample had a shiny metallic surface and exhibited anticipated surface tension interactions with respect to the Al_2_O_3_ crucible. XRD and EDS analysis of the samples did not indicate the presence of other compounds or elements other than those placed in the sample volume. As such, the origin of this peak cannot be identified and is hypothesized to be a tool artifact or reaction occurring outside the sample volume. The unidentified peak is unique to systems with Al_2_O_3_ in direct contact with In and is not a feature of the measurement of In contained in the Mo crucible suggesting that it is unrelated to any interactions of TZM constituents with In and can be considered to be a part of the base line.

Upon removal of the reference In-Al_2_O_3_ background signal from the In-carbide traces, no statistically significant deviations or peaks can be identified. The finding for non-interaction of In with TiC is supported by Kononnako et al. who suggested that TiC is resistant to In up to 847 °C based on the contact angle [28].

Review of the samples post run, the In had formed into a single drop having a surface which was dark grey, similar to the color of TiC and ZrC powder. The droplet was sur-rounded by and in contact with TiC and ZrC powder which reveals a non-wetting behavior which is described in literature for In and TiC [28]. These observations indicate that In was in contact with the carbides during the run, however, they separated despite being intermixed pre run. This is not surprising as In is not known to significantly react with carbides, form its own carbide or dissolve carbon into its melt as evidenced by the lack of a reported In-C phase diagram [27].

Based on this study, no detectable chemical reactions were observed between In and the constituents of TZM. This suggests no adverse chemical compounds are anticipated to form in or on TZM samples when exposed to In that would interfere with the precipitation hardening effects of the carbides in TZM.

### 3.3. TZM-Indium Interface

While the DTA study suggested no compound formation between In and the individual TZM constituents, it is unable to determine the ability for In to diffuse into or otherwise interact with a bulk TZM sample containing all the constituents simultaneously. The possible diffusion of In into the bulk or grain boundaries to TZM is hence investigated using EDS and SEM via samples which were exposed to In and then cross sectioned to determine any interactions. The metallographic investigation already indicated a lack of significant dissolution of TZM into In and proper determination of the TZM-In interface was hence possible.

**Figure 5:**
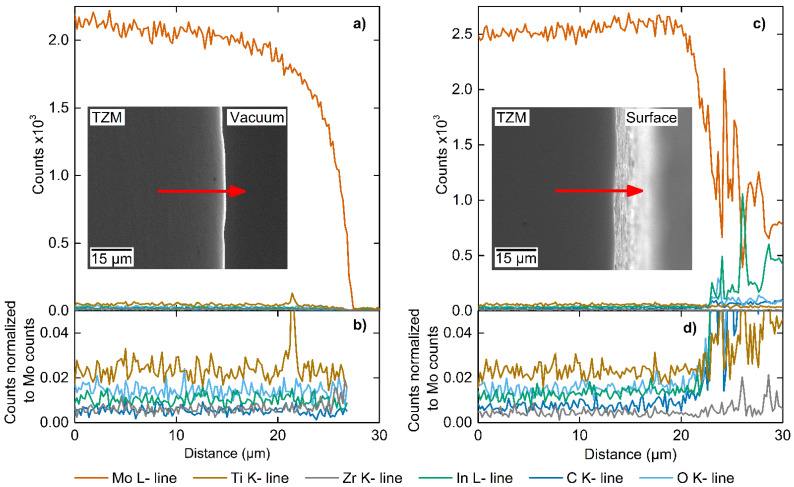
Elemental distribution within a TZM cross-sections exposed to In at 750 °C for 14 days, measured (**a**,**b**) perpendicular to the metallographic prepared surface and (**c**,**d**) tilted to acquire signals from the metallographic prepared and unprepared surface. Inserts show SEM images with EDS line scan directions indicated by an arrow. Panel (**b**,**d**) profile close-up view of the low-count traces (<200 counts) normalized to respective Mo matrix counts.

EDS line scans of a representative polished In-exposed TZM cross sectioned sample are shown in Figure 5. The results were obtained from a TZM sample which was exposed to In at 750 °C for 14 days. Solidified In from the melt was carefully removed prior to measuring the EDS profile to minimize spurious signals from pure In, along with the challenge of polishing the sample due to the soft In embedding particles from grinding. SEM and LOM investigations of the samples did not reveal evidence for dissolution of the TZM surface in liquid In and hence the original TZM-In interface was retained.

Figure 5a shows the recorded elemental distribution from the bulk of the TZM sample across the TZM-In melt interface. The Mo signal drops precipitously upon approaching the interface due a decreased interaction volume of the electron beam within the TZM sample and finally drops to zero after passing the edge of the sample into vacuum.

Figure 5b shows a close-up of the low-count signals after normalizing them to the Mo matrix signal count. The concertation profiles for In, Ti, Zr, C, and O are relatively constant up the interface suggesting no preferential loss of elemental species to the In melt or the inward diffusion of In into the sample. The small spike in the Ti count is due to the presence of a TiC particle close to the interface. Given typical sensitivities of EDS down to 1–2 wt%, these signals can be considered at or below the noise/detection level. The higher level of Ti relative to Zr is in agreement with the higher alloy concentration of Ti in TZM. No In was detected in the bulk or close to the interface of the sample.

The elemental profile shown in Figure 5c was recorded after tilting the same sample such that the as-cut, In-exposed, cleaned, yet not metallographic prepared surface was revealed. The new scanning path was set to acquire signals from the bulk, the interface and the In-exposed surface region from a different equivalent region.

Indium was not detected in the bulk or close to the interface. After passing the interface, and hence measuring signals from the metallographic unprepared surface, the concentration of C, O, and In raised above the detection limit of the system. The detected In is hence on the surface of the sample and did not measurably penetrate into the TZM sample. Figure 5d illustrates the concentration profiles of In, Ti, Zr, C, and O normalized to Mo. All elements show constant signals in the bulk of the sample and all but Zr are increasing sharply after passing the interface.

XRD analysis of the cross-sectional area of the TZM sample, confirmed the exclusive presence of Mo and hence a lack of In. TiC and ZrC were not observed to their low concentrations in agreement with literature [35].

The observed, small amounts of excess In on the unprepared surface, when integrated over the entire surface of the sample, can account for the measured mass gain of 0.009% for the sample further supporting the argument of minimal dissolution of Mo into the static In melt.

The measured rise of the O signal on the surface of the sample is due the formation of a thin surface oxide layer as no increase in the O signal is measured directly at the interface (Figure 5a). The oxide layer is composed of In_2_O_3_ and/or In_2_(MoO_4_)_3_ which would have formed in trace amounts during the post-run processing of the sample in slightly impure nitrogen gas. XRD scans of the TZM surface did not indicate the presence of a molybdenum oxide film. Given sufficient solubility of O in liquid In (~0.33 at% at 1000 °C) [36] to dissolve trace amounts of In_2_O_3_ and decomposition of In_2_(MoO_4_)_3_ above 935 °C into In_2_O_3_ and volatile MoO_3_, a bare, oxide-free surface is anticipated during the high temperature In exposure runs [13,37]. Given comparable EDS line scan and microstructural analyses for the 750 and 1000 °C samples, the presence of a thin protective oxide layer during the run is hence considered improbable. Indium would therefore have likely been in direct contact with TZM during the duration of the test and not moderated by a thin layer of In_2_(MoO_4_)_3_.

Based on the EDS characterization, it is possible to calculate an upper bound for the diffusion coefficient of In into TZM assuming a detection limit of ~1%. Assuming an EDS interaction area of 1 µm^2^ and an uncertainty in measurement close to the interface of 1 µm, a diffusion coefficient of In into bulk TZM must be smaller than 4.1 × 10^−19^ m^2^ s^−1^ at 1000 °C.

### 3.4. Mechanical Properties

#### 3.4.1. Tensile Properties

No measurable In diffusing into the bulk material of TZM was observed. Nevertheless, it cannot be ruled out that trace amounts of In diffused into the grain boundaries. Indium alloy formation in the grain boundaries is anticipated to weaken the material and negatively impact its strength and fracture toughness. Even in small concentrations liquid embrittlement can be detrimental as evidenced by liquid embrittlement of aluminum by gallium [38].

**Figure 6:**
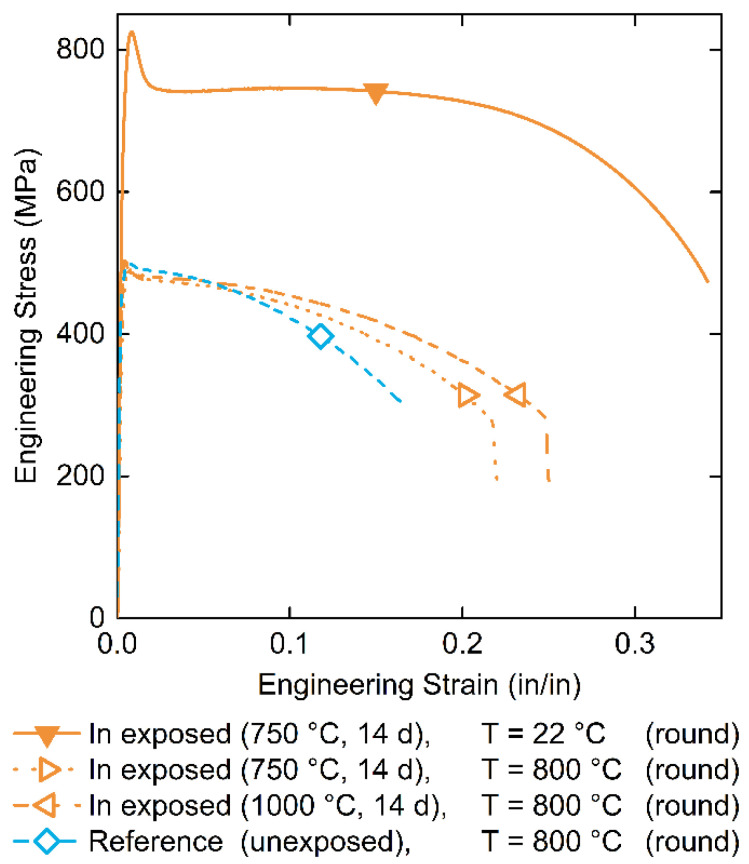
Engineering stress–strain curves of TZM tensile samples from this study: Samples exposed to In at 750 °C (▼ and ▷) and 1000 °C (◁) for 14 days are reported in orange, while the reference, unexposed sample (◇) is blue. Closed symbols indicate room temperature testing, while open symbols at 800 °C. All samples were round tensile specimens (round).

TZM tensile specimens were prepared and exposed to In at 750 and 1000 °C. Post-run and cleaning, they were then pulled at room temperature and at 800 °C. Reference tensile specimens from the same material stock were pulled at the same temperatures. Resulting engineering stress–strain curves are reported in Figure 6 and extracted mechanical properties are provided in Table 2.

Indium-exposed and unexposed TZM tensile samples pulled at 800 °C show comparable behavior and strength values. The total elongation at failure of the reference samples pulled at 800 °C range from 17–32% thereby bracketing the values for the In-exposed samples at 750 and 1000 °C with an elongation of 22% and 25%, respectively.

The observed trend for decreasing elongation and constant reduction in area (RA) with increasing pulling temperature has also been observed in the literature [39,40]. Elongation at failure decreases with increasing temperatures above 100 °C, whereas, the reduction in area is largely unaffected by the test temperature.

Stress–strain curve measurements of unexposed TZM at room temperature and 400 °C are similar to data reported by Filacchioni et al. (2002) [33]. Interestingly, the Young’s modulus reported by Filacchioni et al. for TZM is an outlier from the typically observed values for TZM. No reason was provided for this deviation, though, it should be mentioned that it can be challenging to accurately measure the Young’s modulus using tensile testing due to the need of a high accuracy extensometers which may not have been used [41]. Due to this uncertainty, while presumably isolated to the elastic region, nonetheless, should be applied to all mechanical property data reproduced from Filacchioni et al. in this study.

**Figure 7:**
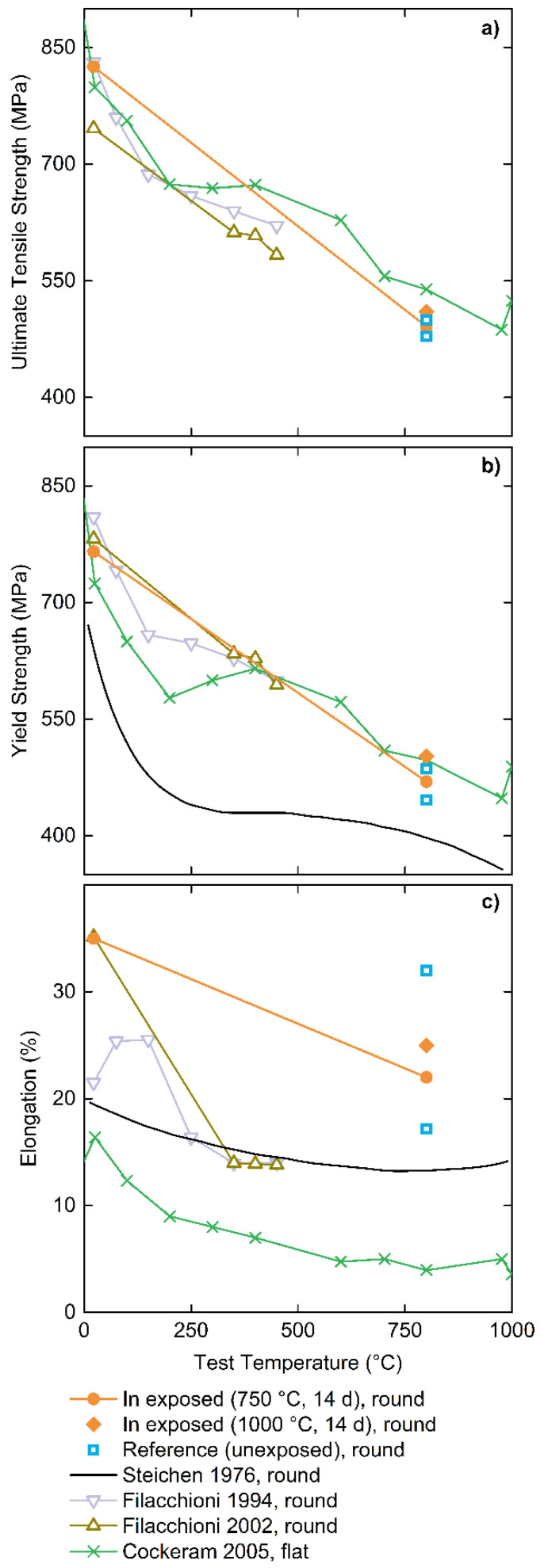
Summary of tensile properties (**a**) ultimate tensile strength, (**b**) yield strength and (**c**) elongation at failure) for sample temperatures ranging from 0–1000 °C. Solid symbols indicate TZM samples exposed (●,⬥) and not exposed (□) to In from this study while open or no symbols are literature data (△, ▽, x) [33,39,40,42] Tensile specimen geometry is either round or flat. Lines connecting symbols are guides for the eye only while the symbol-free trace is from continuous data sampling.

To evaluate the change in tensile properties as a function of operating/testing temperature, Figure 7 summarizes (a) the ultimate tensile strength, (b) the yield strength and (c) the total elongation for samples pulled in this study and literature reported values [33,39,40,42]. All TZM materials shown in Figure 7 were tested in the stress-relieved condition and pulled in the longitudinal direction. All TZM materials were formed from powder metallurgy processed raw materials with the exception of Steichen (1976) [42] and Cockeram (2005) [39], which were manufactured by vacuum arc-casting. Filacchioni et al. (1994) [40] did not report their TZM forming method. It is also important to note that the curves reported by Cockeram (2005) [39] were acquired by measuring samples machined from plates which are known to generally show different tensile characteristics.

Ultimate tensile and yield strength measured in this study generally compare congruently, when considering anticipated and common statistical variations for these types of samples and measurements, to literature data at applicable temperatures. Exposure of TZM to In leads to a statistically insignificant deviation from the reference samples part of this study. The exposure of In to TZM has no measurable negative influence on the mechanical behavior up to the investigated 1000 °C soak temperature. Conversely, the obtained tensile data shows higher stress and elongation values for the sample soaked at higher temperatures.

The elongation at failure value for the room temperature test (Figure 7c) matches the value reported by Filacchioni et al. (2002) [33]; however, these values [33] are 10% above other reported literature values [39,42]. Published elongation data decreases strongly with increasing test temperature, while results from this study indicate a weaker decrease in elongation providing a higher ductility at higher temperatures. No definitive explanation can be provided as to why the elongation values of the tested TZM surpasses literature data by such a notable amount and warrants further investigation. It cannot be ruled out that improved processing of the TZM material as compared to literature data and samples has led to this behavior.

As the tensile samples did not fail brittle at room temperature, it can be stated that the low strain rate ductile to brittle transition temperature (DBTT) occurs below room temperature.

In summary, exposure to In did not have any statistically relevant impact (positive or negative) on the yield strength, ultimate tensile strength and elongation behavior of TZM when pulled in tensile testing. Given the lack of noticeable changes to the tensile behavior of TZM, In did not diffuse substantially into the bulk of the TZM specimen even after high temperature exposure for 14 days. These observations suggest TZM is an excellent high strength at high temperature material, even in the presence of In.

#### 3.4.2. Fracture Toughness

**Figure 8:**
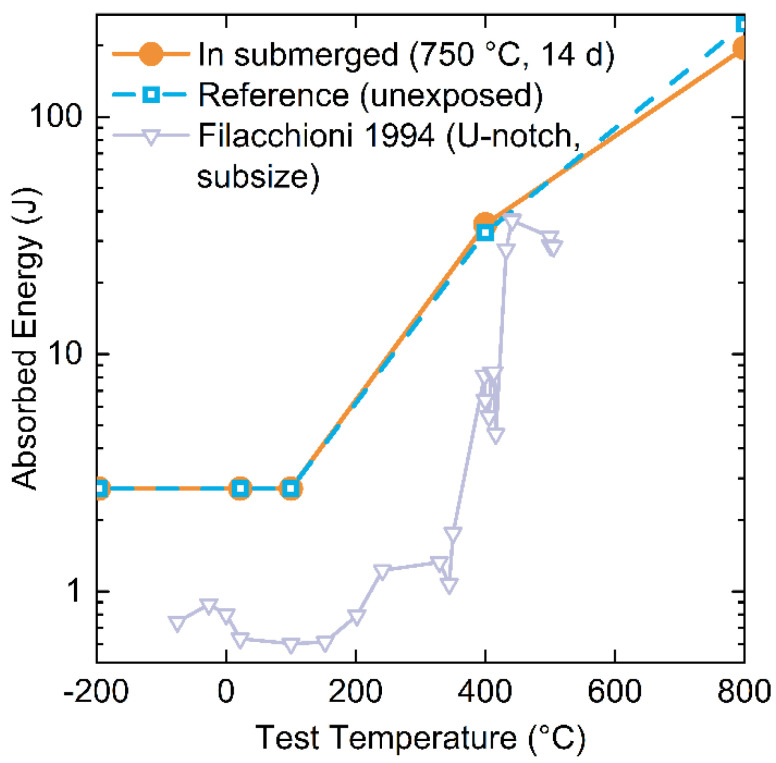
Fracture toughness values for TZM samples in this study for In-exposed (●) and unexposed (□) TZM using standard size Charpy impact test specimens at various sample temperatures. Trianlge (▽) is data from Filaccioni et al. using sub-size U-notch samples [40].

Tensile testing results suggest that there are no significant differences in crack propagation between In-exposed and unexposed TZM samples. Charpy impact tests of In-exposed and unexposed samples were performed to investigate a potential influence of In on the fracture behavior of TZM. Figure 8 shows the results of the impact testing at various testing temperatures.

There is no statistically relevant difference between the measured fracture energy values between TZM exposed to In at 750 °C for 14 days and the unexposed, reference samples. Therefore, it can be concluded that even if In diffuses along the grain boundaries or into the material in general, the diffusion within 14 days is insignificant and does not impact the fracture toughness of TZM.

Comparison to literature data is challenging due to the significant impact of sample geometry and strain rates on reported results. No studies were found to have tested the same sample geometry as reported in this study. The closest geometry was reported by Filacchioni et al. (1994) [40] who used a sub-sized samples and a U-shaped notch (DIN 50115, 6 × 6 × 44 mm). Fracture toughness data obtained by Filacchioni et al. [40] is shown in Figure 8 and exhibits the typical S-shaped curve. Reported data is not normalized to area and hence exhibits lower absorbed energies due to a smaller cross-sectional area for fracture as compared to samples in this study. Qualitatively, data from the literature follows the observed trend in increasing fracture toughness with increasing sample temperature, as anticipated, though differs in absorbed energy due to differing sample geometry.

An important material property which can be gleaned from fracture toughness is the DBTT, though this value critically depends on the strain rate the sample experiences [43]. For Charpy samples (high strain rate) in this study, a DBTT between 100 °C and 400 °C was observed, while the tensile specimen samples (low strain rate) always exhibited ductile behavior, hence they must have a DBTT below room temperature. An equally wide range of DBTTs is reported in the literature, from −85 to 420 °C, suggesting TZM samples from this study exhibit typical TZM behavior [39,40,42,44].

## 4. Summary

This study investigated the consequences of exposing TZM to In from a corrosion and structural perspective. TZM samples were soaked in molten In at high temperature (750 °C or 1000 °C) and then analyzed for metallurgical/chemical changes, compound formation, and mechanical properties changes. The mechanical properties were tested at a range of temperature: Tensile properties were determined at room temperature and 800 °C, while fracture toughness was determined at multiple temperatures between −196 °C and 800 °C. Reference TZM samples, which were not exposed to In and machined from the same stock material, were tested under identical conditions, and used to assess deviation for the In-exposed samples. The following conclusions can be drawn from this study:

1. The TZM microstructure, as analyzed by LOM and SEM, did not reveal any changes or detrimental corrosive interactions with the constituents of TZM. No surface corrosion or other interactions with In were observed in the samples.
2. No compounds were found to form between In and pure Mo, TiC, or ZrC at temperatures up to 900 °C using DTA and XRD.
3. Chemical analysis of the TZM-In interface using EDS and XRD indicated no In diffused into the TZM sample. An upper bound for the diffusion coefficient was estimated to be 4.13 × 10^−19^ m^2^ s^−1^ at 1000 °C.
4. Mechanical properties measured using tensile testing and Charpy V-notch bars indicated no improvement or degradation in yield strength, ultimate tensile strength, elongation to failure, and fracture toughness. Anticipated temperature-dependent trends for mechanical properties based on literature observations were followed from samples from this study.

After 14 days of In exposure, TZM appears to be unaffected and resistant to In corrosion or attack. The TZM sample was not visibly or measurably negatively impacted in mechanical performance. As such, while longer term exposures to In, an improved understanding of the possible existence of a surface passivation layer at lower temperature, the impact of surface roughness and/or static vs. flow conditions of In on the dissolution kinetics of TZM, and creep studies are needed to fully assess the impact, it currently appears TZM is a promising material that can be used as a high strength, high temperature material in the presence of In.

## Figures and Tables

**Table 1:** Chemical compositions in wt% reported by vendor for TZM material.

Sample	Mo	Fe	Ni	Si	C	O	N	Ti	Zr
TZM 1	Balance	<0.001	0.002	<0.001	0.025	0.0191	0.002	0.47	0.085
TZM 2	Balance	0.0008	0.0008	0.0007	0.027	0.003	0.002	0.53	0.08

**Table 2:** Tensile data for TZM samples pulled along longitudinal direction.

Pulling Temperature (°C)	Indium Exposure	Ultimate Tensile Strength (MPa)	Yield Strength (MPa)	Total Elongation (%)	Reduction of Area (%)
22	750 °C, 14 d	825 ± 8	763 ± 8	35.0 ± 0.4	70.0 ± 0.7
800	750 °C, 14 d	491 ± 5	470 ± 5	22.0 ± 0.2	71.5 ± 0.7
800	1000 °C, 14 d	510 ± 5	502 ± 5	25.0 ± 0.3	73.5 ± 0.7
800	unexposed	479 ± 5	446 ± 4	32.0 ± 0.3	75.5 ± 0.8
800	unexposed	499 ± 5	485 ± 5	17.2 ± 0.2	71.9 ± 0.7

## Data Availability

The data presented in this study are available on request from the corresponding author.
